# Integrons in *Salmonella* Keurmassar, Senegal

**DOI:** 10.3201/eid1007.030666

**Published:** 2004-07

**Authors:** Amy Gassama-Sow, Awa Aïdara-Kane, Nabil Raked, François Denis, Marie-Cécile Ploy

**Affiliations:** * Laboratoire de Bactériologie-Virologie-Hygiène, Limoges, France;; † Institut Pasteur, Dakar, Sénégal

**Keywords:** letter, integron, *Salmonella*, new serovar

**To the Editor:** Infections caused by *Salmonella* are the primary cause of foodborne diseases; multidrug resistance to *Salmonella enterica* subsp. *enterica* is increasing. The selective pressure created by the widespread use of antimicrobial agents in animals and humans as prophylactic and therapeutic agents may have contributed to the dissemination of resistant bacterial strains. In 2000, the new serovar Keurmassar (35:c:1,2) of *S. enterica*, was described in Senegal (1). Integrons are efficient gene-capture systems by site-specific recombination and are involved in antimicrobial-drug resistance in gram-negative bacteria (2). Three classes of integrons are well characterized and are involved in antimicrobial resistance. Integrons have been found in different nontyphoidal serovars of *S. enterica* and recently in serovar Typhi (3).

We evaluated the contribution of integrons to the antimicrobial drug resistance of eight isolates of *S. enterica* serovar Keurmassar sent to the Senegalese National *Salmonella* and *Shigella* Reference Laboratory at the Pasteur Institute in Dakar from March to May 2000. One strain was isolated from poultry flesh, and seven strains were isolated from human stool or blood samples. Susceptibility testing was performed by disk diffusion method on Mueller-Hinton agar according to the Comité de l'antibiogramme, Société Française de Microbiologie, recommendations. The eight strains expressed an extended-spectrum β-lactamase, which was previously identified as SHV-12 (1). The strains were also resistant to aminoglycosides (amikacin, gentamicin, netilmicin, spectinomycin, streptomycin, and tobramycin), chloramphenicol, sulfamethoxazole, tetracycline, and trimethoprim. Genomic diversity was studied by pulsed-field gel electrophoresis (PFGE) and analysis of *Xba*I restriction fragments as described previously (3). The eight strains isolated from poultry and humans specimens showed identical PFGE patterns, which suggested that all strains of *S. enterica* serovar Keurmassar isolated until May 2000 in Senegal belonged to the same clone.

Strains were screened for the integrons by polymerase chain reaction by using three sets of primers specific for the *intI1*, *intI2*, and *intI3* genes coding for the integrase as described previously (3). The *intI1* gene was detected in all strains. Class 2 or 3 integrons were not detected. Cassette assortment in class 1 integrons was determined by using the primers 5´CS and 3´CS complementary to the 5´ and 3´ segments as described previously (3). With these primers, we obtained two amplification products of 1 kb and 1.7 kb for each strain, which suggested that all strains contained at least two class 1 integrons. Sequencing of these amplification products showed that the first product of 1 kb contained the *aadA2* cassette, which confers resistance to streptomycin and spectinomycin. The second amplicon of 1.7 kb carried a new arrangement of two cassettes: *aac(6´)-IIc*, which confers resistance to gentamicin, netilmicin, and tobramycin; and *ereA2*, which encodes resistance to erythromycin. Class 1 integrons were previously found in strains of *S. enterica* of different serovars: Agona, Albany, Brandenburg, Enteritidis, Goldcoast, Hadar, Infantis, Ohio, Panama, Poona, Saintpaul, Typhi, Typhimurium, Virchow, and Worthington (3–7). All these integrons, except that of serovar Infantis, contained a streptomycin-spectinomycin resistance determinant, *aadA2* or mostly *aadA1*, alone or in combination with other gene cassettes. The cassette *aac(6´)-IIc* was previously described in a single class 1 integron in *Pseudomonas aeruginosa* (AF162771). The *ereA2* cassette was first described in *Providencia stuartii* (8) in a class 1 integron. This cassette was since described in class 1 integrons of clinical gram-negative isolates and recently in a class 2 integron in *Escherichia coli* (9). To determine whether the resistance determinants carried by the integrons were transferable, we performed a conjugation experiment from *S. enterica* serovar Keurmassar to an *E. coli* strain resistant to nalidixic acid. We first used a selective medium containing nalidixic acid 50 µg/mL plus 25 µg/mL of streptomycin, one of the two integrons carrying the *aadA2* cassette. All antimicrobial drug resistances were transferred at once from each strain to *E. coli*. The analysis of plasmid content prepared by alkaline lysis method from all transconjugants showed a single plasmid of >30 kb. The polymerase chain reaction analysis of the plasmid DNA confirmed the transfer of the two integrons, which suggested that the integrons were borne by a conjugative plasmid.

The multidrug resistance of these strains could be explained by the fact that antimicrobial agents are used extensively in the poultry industry in Senegal to reduce deaths and to increase productivity (1). Moreover, in Senegal, as in many countries in Africa, antimicrobial agents are sold over the counter, which leads to self-medication, thus increasing the selective pressure.

This is the first finding of integrons in the newly described serovar Keurmassar of *S. enterica*. One integron contained two cassettes, *aac(6´)-IIc* and *ereA2*. This was also the first finding of such an integron with a new arrangement of these two cassettes in a clonal strain of *S. enterica* serovar Keurmassar that had recently emerged. Indeed, the *aac(6´)-IIc* cassette was described only once in *P. aeruginosa*. Moreover, aminoglycosides are not used extensively in Africa because they are very expensive. Therefore, determining how this cassette combination was selected is difficult. The strains studied were resistant to multiple antimicrobial agents, including broad-spectrum cephalosporins by production of the extended-spectrum β-lactamase SHV-12. The two class 1 integrons described here could account for the resistance to only a few drugs. The *bla*_SHV-12_ gene was not carried by an integron. Otherwise, ampicillin, trimethoprim, and tetracycline are the antimicrobial agents commonly used to treat diarrheal diseases in Africa. All of the strains studied were resistant to these antimicrobials agents. Trimethoprim resistance *dfr* genes are frequently found in integrons (2). However, in this study, we were not able to detect integrons containing *dfr* cassettes.

The presence of a conjugative plasmid and integrons in this serovar is of clinical importance. Indeed, the spread of the multiple antimicrobial agent resistance to other *Salmonella* serovars or gram-negative bacteria might easily occur by the transfer of such a plasmid. Moreover, integrons could allow the acquisition of new genes.
